# Improving diagnosis and support for people with bipolar in primary care

**DOI:** 10.3399/BJGP.2025.0709

**Published:** 2026-06-01

**Authors:** Victoria Silverwood, Thomas Round, David Kessler, Simon Kitchen, Carolyn A Chew-Graham

**Affiliations:** 1 School of Medicine, Keele University, Staffordshire, UK; 2 King’s College London, London, UK; 3 Primary Care, Bristol Medical School, University of Bristol, Bristol, UK; 4 Bipolar UK, London, UK

## What is bipolar?

Bipolar is a lifelong, severe mental illness characterised by mood swings and fluctuating energy levels. Clinicians may be more familiar with the term ‘bipolar disorder’; however, bipolar is the current favoured term with patient groups^1^ and will be used throughout this article.

People with bipolar may have periods of mood stability with long periods of full or partial wellness, before experiencing severe depression (‘lows’), or hypomania, mania, or psychosis (‘highs’).^1^ The most common types of bipolar are bipolar type 1 (bipolar1), bipolar type 2 (bipolar2), and cyclothymia. Lifetime prevalence of bipolar, including cyclothymia, is estimated to be 2.4%, with estimated aggregate lifetime prevalence of bipolar1 and bipolar2 being between 0.6–1.1% and 0.4–1.6% respectively.^2,3^ Prevalence might be higher among certain groups – for example, the estimated prevalence of bipolar in homeless people is 11.4%.^4^

Bipolar1, considered the most severe type of bipolar, is defined by the presence of at least one manic episode alongside periods of depression.^5^ Bipolar2 is characterised by an episode of hypomania alongside depressive episodes and often poses a greater diagnostic challenge, as hypomanic episodes may be short lived and are not as easily recognised.^5^ Cyclothymia is characterised by symptoms of hypomania and low mood that that do not meet the diagnostic criteria for Bipolar1 or Bipolar2.^6^ Mood disturbances in cyclothymia can rapidly cycle, with fleeting oscillations between euphoria and depression, sometimes resulting in a misdiagnosis of personality disorder.^7^

Bipolar can result in significant morbidity; people with bipolar have a reduced life expectancy of up to 15–20 years, predominantly due to increased risk of cardiometabolic disease.^8^ People with bipolar are more likely to engage in risky behaviours, have problems with interpersonal relationships, and are at increased risk of dying by suicide.^9^

## When should bipolar be suspected in primary care?

Most patients with bipolar have their first episode of depression or mania/hypomania before the age of 25 years; however, it is recognised that there is often a delay of up to 9 years from first presentation of symptoms until diagnosis.^10–12^ The exact pathogenesis of bipolar is not fully understood; risk factors include genetic susceptibility, a family history of bipolar, exposure to adverse and traumatic childhood events, and excessive alcohol use.^9^

Many patients with bipolar are diagnosed with depression and might be labelled as having ‘treatment- resistant depression’ when antidepressants do not improve their mood.^13^ To avoid missing a potential diagnosis of bipolar, National Institute for Health and Care Excellence (NICE) clinical guidance^14^ recommends that all patients who present with depression should be asked about the possibility of previous manic or hypomanic episodes by enquiring about periods of overactivity or disinhibited behaviour. Box 1 outlines scenarios when GPs could consider bipolar as a potential diagnosis.

**Box 1.** When should GPs consider bipolar?

Patients prescribed multiple psychotropic medications who have not seen any benefit. Patients diagnosed with other mood disorders such as depression who repeatedly engage in risky behaviours. Patients with a family history of bipolar. Patients with a pattern of escalating self-harm. Patients with increasing frequency of presentations to healthcare services. Patients with issues such as sleep disturbance/insomnia, substance misuse, or suspected personality disorder coded in their notes.

GPs should refer patients urgently to psychiatry if a patient has had disinhibited behaviour or overactivity for over 4 days at a time, if mania or severe depression is suspected, or if a patient poses a risk to themselves or others.^14^ Patients can complete the Bipolar UK (www.bipolaruk.org.uk) mood diary to send with the referral as supporting information.^1^ Depending on local provision for accessing urgent secondary care psychiatric review, GPs may be able to access psychiatric advice for support while patients are awaiting assessment.

## Management of people with bipolar

Management of people with bipolar1, bipolar2, and cyclothymia should be a multifaceted approach across primary and secondary care, including medication, psychological therapies, management of physical health, and support for families and carers.

### Medication

Traditionally, first-line therapy for bipolar (including cyclothymia) includes mood stabilisation medications such as lithium and atypical antipsychotic drugs such as olanzapine, quetiapine, and aripiprazole.^7,9^ Adherence to medication can be challenging as many patients experience side effects such as flattened mood, anhedonia, and weight gain.^9^ Previously, sodium valproate was more commonly used; however, because of known side effects and high rates of teratogenicity, in 2019 the Medicines and Healthcare products Regulatory Agency issued guidance restricting the use of sodium valproate in women <55 years unless two specialists agreed that this was the preferred treatment option,^15^ extending the restriction to men <55 years in 2024.^15^

Long-term use of antidepressant medication such as selective serotonin reuptake inhibitors (SSRIs) is generally not recommended when patients are stable, because of the risk of inducing mania. However, recent evidence suggests that there may be a role for a combination of an atypical antipsychotic and an SSRI for some patients during depressive episodes.^16^ Medications to treat bipolar should be initiated in secondary care and, once the patient is stable, GPs can continue to issue them as part of a shared care agreement.^14^

### Talking therapies

Patients with bipolar and cyclothymia can benefit from talking therapy services for psychological interventions alongside medications.^14^ Therapy options include cognitive behavioural therapy, interpersonal therapy, family-focused therapy, and group psychoeducation programmes.^17,18^

### Support for families and carers

As well as social support for patients with bipolar, it is important to consider how to support family members and carers. GPs can note these family members as carers in the patient’s medical records and offer them an annual review, referring on to services such as social prescribing for support as needed.^19^ Patients, as well as family members and carers, may find the Bipolar UK website (www.bipolaruk.org) helpful for information and support.

## How should general practice support people with bipolar?

Once patients have received a diagnosis and started treatment in secondary care, patients may be discharged to primary care for ongoing management and support. It is helpful to document a relapse management plan for patients with bipolar in their medical records. This provides a helpful prompt for clinicians unfamiliar with the patient to be aware of their warning signs of relapse. GPs should refer patients with a bipolar diagnosis who present with signs of relapse to acute mental health services for urgent review. GPs may be the first clinicians to identify side effects from psychiatric medication at medication reviews. If side effects are identified that might affect continuation of those medicines, these can then be highlighted urgently to the secondary care psychiatry team.

Patients with bipolar should be added to practices’ Severe Mental Illness (SMI) register and receive an annual physical health review appointment. Previously, establishing and maintaining this register was included as part of the Quality and Outcomes Framework (QOF) within primary care, therefore attracting financial reimbursement. As of April 2025, this QOF requirement was removed.^20^ Despite this, it is crucial that GPs continue to monitor and support the physical health of patients with bipolar.

**Figure 1.**
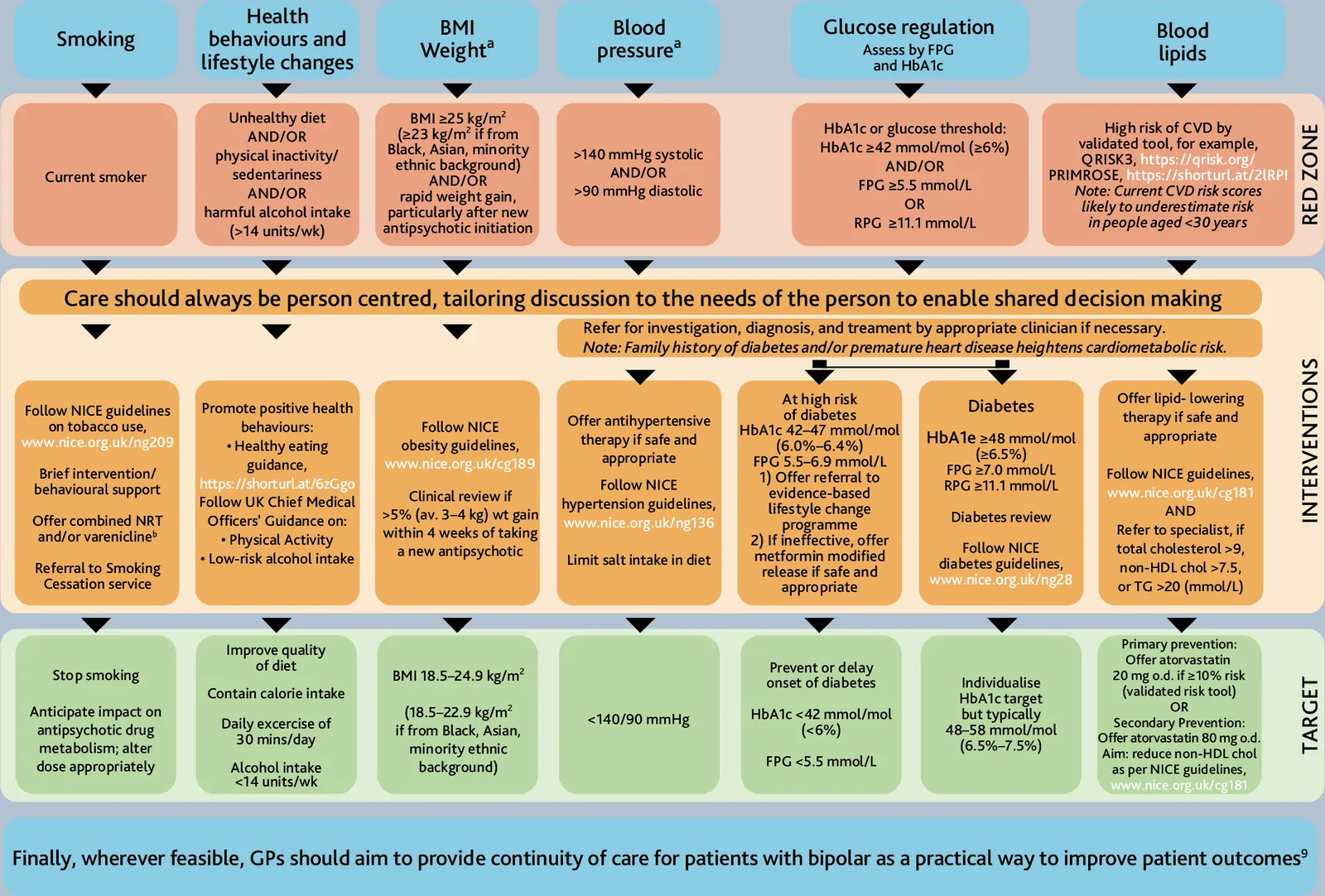
Positive cardiometabolic health resource: an intervention framework for people experiencing psychosis and schizophrenia. Adapted from the Lester resource.^8,14^ © Royal College of Psychiatrists. ^a^Developmentally appropriate norms should be used for people aged <18 years. ^b^The pharmacy should be consulted to check local availability. BMI = body mass index. Chol = cholesterol. CVD = cardiovascular disease. FBG = fasting blood glucose. HDL = high-density lipoprotein. NICE = National Institute for Health and Care Excellence. NRT = nicotine replacement therapy. o.d. = once a day. RPG = random plasma glucose. TG = triglycerides.

The Positive Cardiometabolic Health Resource in Figure 1 ^8^ provides practical guidance for annual review appointments for all patients with SMI. This should include measurement of blood pressure, body mass index, and appropriate bloods (for example, lipid profile, HbA1c). For those on medication monitoring, other bloods such as full blood count, urea and electrolytes, and lithium levels might be appropriate every 6–12 months. Patients should be offered lifestyle advice, smoking cessation support where indicated, and can be reminded about the importance of dental health check-ups.^21^ It is also an opportunity to consider screening programmes that patients may have missed out on such as cervical, breast, and bowel screening.

## Key messages for primary care

- Bipolar is a lifelong SMI, associated with a high risk of morbidity and reduced life expectancy.^8^
- Early diagnosis and intervention are key.
- Management of bipolar should be a multifaceted approach across primary and secondary care, including medication, psychological therapies, management of physical health, and support for families and carers.^9,14,16^
- GPs should ask all patients who present with depression about the possibility of previous manic or hypomanic episodes by enquiring about periods of overactivity or disinhibited behaviour, and ask about any family history of bipolar.
- GPs play an important role in providing holistic care, supporting both mental and physical health for people with bipolar via regular primary care reviews.^22^
- Awareness of local referral systems for initial diagnosis and crisis support is crucial.
- Where feasible, GPs should aim for continuity of care with one clinician, with a clear relapse plan documented in a patient’s medical records.^9^
- GPs can direct patients and their families towards services such as www.bipolaruk.org for information and support.^1^
